# Light-Driven Quantum Dot Dialogues: Oscillatory Photoluminescence in Langmuir–Blodgett Films

**DOI:** 10.3390/nano15141113

**Published:** 2025-07-18

**Authors:** Tefera Entele Tesema

**Affiliations:** Department of Chemistry, Prairie View A&M University, Prairie View, TX 77446, USA; tetesema@pvamu.edu

**Keywords:** quantum dots (QDs), Langmuir–Blodgett films, PL, interdot coupling effects, oscillatory PL dynamics, interlayer atom transfer

## Abstract

This study explores the optical properties of a close-packed monolayer composed of core/shell-alloyed CdSeS/ZnS quantum dots (QDs) of two different sizes and compositions. The monolayers were self-assembled in a stacked configuration at the water/air interface using Langmuir–Blodgett (LB) techniques. Under continuous 532 nm laser illumination on the red absorption edge of the blue-emitting smaller QDs (QD450), the red-emitting larger QDs (QD645) exhibited oscillatory temporal dynamics in their photoluminescence (PL), characterized by a pronounced blueshift in the emission peak wavelength and an abrupt decrease in peak intensity. Conversely, excitation by a 405 nm laser on the blue absorption edge induced a drastic redshift in the emission wavelength over time. These significant shifts in emission spectra are attributed to photon- and anisotropic-strain-assisted interlayer atom transfer. The findings provide new insights into strain-driven atomic rearrangements and their impact on the photophysical behavior of QD systems.

## 1. Introduction

Since their initial use in light-emitting diodes (LEDs) by Colvin et al. [1], semiconductor quantum dots (QDs) have become essential in optoelectronics due to their size-tunable, narrow, and bright emission. They enable high-performance displays—such as QD color filters and ultra-high-display screens [2]—and efficient QD-LEDs, with external quantum efficiency (EQE) exceeding 18% through optimized architectures [3]. In photovoltaics, PbS and CdSe QDs offer tunable bandgaps and multiple exciton generation, achieving over 15% efficiency in third-generation solar cells [4,5]. Their stable fluorescence and high surface area make QDs ideal for chemical sensing via FRET, PET, and charge transfer, with CdTe QDs detecting Cu^2+^ at trace levels [6]. In biomedicine, QDs pioneered by Bruchez et al. and Chan & Nie [7,8] are widely used for labeling, imaging [9,10], and diagnostics, including sensitive detection of biomarkers like miRNA−21 linked to cancer and psychiatric disorders [11], positioning QDs as versatile platforms across advanced technologies [12,13,14,15].

However, the conventional binary QDs have size-dependent emission properties, which restricts independent control over optical wavelength and particle size. Alloyed QDs, such as CdSeS and CdSeTe, provide a solution to this limitation by offering bandgap tunability via compositional adjustment at a fixed size. This decoupling is particularly valuable in applications requiring monodisperse particle size with precise optical outputs. Chen et al. showed that CdSeS-alloyed QDs could be systematically tuned from green to red emission by adjusting the Se/S ratio while preserving the nanocrystal size and stability [16]. These alloyed systems also exhibit reduced lattice mismatch and improved photostability when protected with suitable shells, like ZnS.

Beyond intrinsic tunability, the optical properties of alloyed QDs are highly sensitive to external environmental conditions. Factors such as substrate interaction, film morphology, interlayer coupling, and ligand integrity can substantially influence emission characteristics. Layer-by-layer (LbL)-assembled films where monolayers of QDs are deposited sequentially onto substrates present opportunities for engineered light–matter interactions but also introduce complexity through interdot coupling, strain accumulation, and photoinduced modifications. Previous studies have shown that photoluminescence (PL) from QDs can shift in wavelength, broaden in linewidth, or fluctuate in intensity depending on excitation power, ambient conditions, or substrate chemistry [7,16,17,18,19,20,21]. We have demonstrated in the past that strain-driven atomic interdiffusion of alloyed quantum dots at interfaces during film formation significantly alters their emission properties, highlighting the importance of post-synthetic stability studies and their implications for long-term optoelectronic and biomedical imaging applications [19]. Yet, detailed investigations into photophysical behaviors and bandgap modulation effects in layered alloyed QDs remain scarce, especially for CdSeS/ZnS core/shell systems under continuous optical excitation.

In this work, we investigate the temporal and spectral evolution of photoluminescence in layered CdSeS/ZnS quantum dot films assembled via Langmuir–Blodgett (LB) deposition on oxide-coated silicon substrates. The layered structure consists of a bottom layer of blue-emitting QD450 (∼9 nm) and a top layer of red-emitting QD645 (∼12 nm), with oleic acid ligands serving as the optically transparent spacer layer. Under continuous 532 nm laser excitation focused on the top layer, we observe an unexpected combination of PL intensity oscillation, progressive blueshift in the peak wavelength, and broadening of the emission linewidth (FWHM). These effects are absent or minimal in control architectures lacking interlayer heterojunctions or compositional asymmetry. Through this, we aim to uncover new insights into the environmental effect on the exitonic behavior of QD heterostructures and inform the design of future photonic, biosensing, and optoelectronic applications using engineered nanocrystal films.

## 2. Materials and Methods

### 2.1. Materials

A 10 mg/mL suspension in chloroform of CdSSe/ZnS or CdSe/ZnS core/shell quantum dots (QDs) consisting of a monolayer of oleic acid coating were obtained from Ocean NanoTech LLC, San Diego, CA, USA, and used as received. The QDs have an emission wavelength peak ranging from 450 nm to 645 nm, with a quantum yield >50%.

### 2.2. Preparation of QD Film by Langmuir–Blodgett (LB) Techniques

Quantum dot thin films were deposited on oxide-coated silicon wafers and cover glass substrates. These substrates were cleaned by sonicating in acetone, isopropanol, and ultrapure water for 5 min each, followed by a 5 min ultraviolet ozone treatment (Novascan Technologies, Inc., Ames, IA, USA). Then, the substrate was then vertically immersed into the ultrapure deionized (DI) water subphase of the Langmuir–Blodgett (LB) trough to a pre-programmed height. Although the water surface was initially clean and stable, the immersion disturbed the subphase and could potentially introduce impurities. Any such impurities were carefully removed, and the system was allowed to re-stabilize before applying sample on subphase. height and the water surface was clean and stable. A total of 10–20 microliters of a 10 mg/mL solution of quantum dots in a chloroform was gently applied to the water surface using a Hamilton microliter syringe and was allowed to stabilize for 20 to 30 min. The film was then compressed at a typical barrier speed of 10 mm/min until a target surface pressure of 20–40 mN/m was attained and a close-packed monolayer film of QDs at the air/water interface was formed. The film was allowed to be stabilized for about 5 min. Then, the film was transferred to a substrate as it moved automatically vertically out of the subphase at a speed of 5 mm/min under a constant surface pressure.

### 2.3. Optical Characterization

A collimated, linearly polarized light from lasers (405 nm and 532 nm diode lasers) was attenuated to a power density of 53 mW/cm^2^ and 55 mW/cm^2^, respectively, on the sample and directed to Olympus GX51 inverted microscope (Olympus Corporation, Tokyo, Japan) and focused on the sample using a 20× objective. The collected PL was passed through the same objective, and the signal was sent to a spectrometer (IsoPlane Spectrograph of Princeton Instruments, Trenton, NJ, USA) with a cooled CCD camera while a long-pass filter removed any remaining source of light. This setup allows for PL collection from a fixed area by switching excitation sources without moving the components or the sample.

### 2.4. Structural Characterization

#### Atomic Force Microscope (AFM)

Atomic force microscope (AFM) images were recorded in air using a Neaspec-GmbH (Haar, Germany) instrument operated in a tapping mode with a tapping amplitude ≈50 nm near the resonance oscillation frequency of the cantilever. The cantilevers used were the NCHR series of standard and supersharp silicon (Nanoworld, NanoAndmore, Watsonville, CA, USA), with a resonance frequency of 250–390 kHz and an average tip radius of ≤5 nm and ≤12 nm for super sharp and standard AFM tips, respectively.

## 3. Results and Discussion

**Figure 1:**
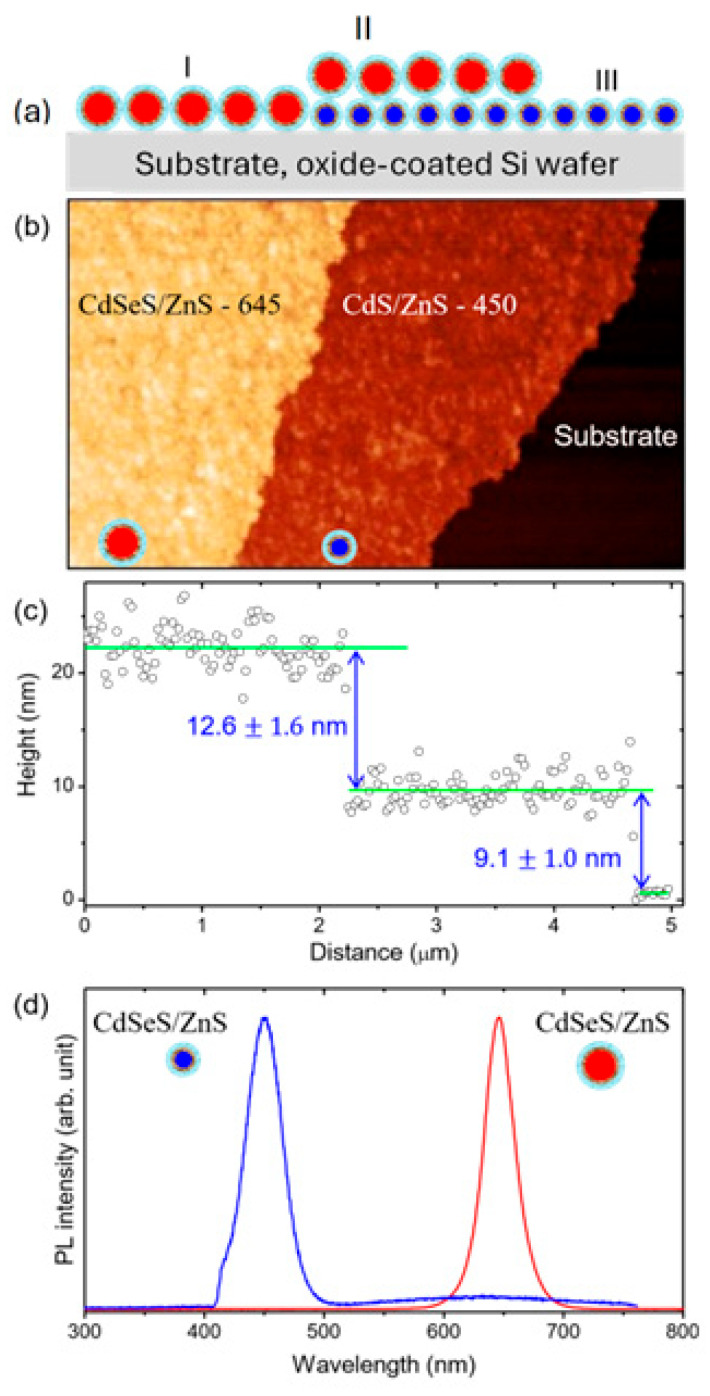
(**a**) Schematic illustration of the layered monolayer assembly of CdSeS/ZnS quantum dots (QDs), showing red-emitting QD645 and blue-emitting QD450 deposited on an oxide-coated silicon substrate. Regions I and III correspond to areas of primarily single-layer QD645 and QD450, respectively, while region II represents the double-layer zone where major photoluminescence (PL) dynamics will be discussed in more detail later in the text. (**b**) Representative AFM topography image of the stacked QD monolayers, indicating the distribution of QD domains. (**c**) AFM height profile across the QD interfaces, showing average monolayer heights of 12.6 ± 1.6 nm for QD645 and 9.1 ± 1.0 nm for QD450. (**d**) Photoluminescence spectra collected from regions I (red line) and III (blue line) as defined in panel (**a**), demonstrating distinct emission peaks associated with the two QD layers.

The structural organization, thickness, and optical properties of the self-assembled QD layers are depicted in Figure 1a–d. The data in the figure serves as a foundational characterization of the LB QD film, demonstrating its structural integrity, precise thickness control, and distinct photoluminescence properties. These attributes set the stage for subsequent investigations into optical coupling as well as the photophysical and photochemical dynamics.

The experimental approach is illustrated in the schematic in Figure 1a. The temporal evolution of the spectral characteristic of the QDs in region II, where a monolayer of red-emitting CdSeS/ZnS QDs (QD645) is formed on top of a monolayer of a blue-emitting CdSeS/ZnS QDs (QD45)), is compared with that of the QDs in regions I and III, where the respective QDs are directly assembled on a silica substrate. A representative atomic force microscope (AFM) topographic image of the layer-by-layer-assembled QD monolayers that are prepared using a Langmuir–Blodgett (LB) procedure is shown in Figure 1b (the details of the procedure are provided in Section 2.2 and Section 2.4). The line profile along the dashed line indicates a thickness of 9.1 ± 1.0 nm and 12.6 ± 1.6 nm for the bottom (blue-emitting) and top (red-emitting) monolayers of the QDs, respectively, as shown in Figure 1c. On the other hand, from the transmission electron microscope (TEM) images (Supporting Information Table S1a and Figure S1a), the average core + shell diameters of the QD645 and QD450 core/shell QDs are determined as 10.2 ± 0.7 nm and 7.0 ± 0.6 nm, respectively. Similarly, Figure S1b (high-resolution TEM (HRTEM)) reveals the core diameter of the bigger and smaller QDs to be 8.0 ± 0.2 nm and 5.5 ± 0.3 nm, respectively. The Se to S mole ratio in the QD645 is ~2:1 compared to 1:5 for the QD450 as estimated from Energy-Dispersive X-ray Spectroscopy (EDS), Figure S1c. Combining the AFM and TEM results, oleic acid surface ligands are expected to provide about 1.5–2.5 nm spacing between the blue- and red-emitting QD monolayers. The peak emission wavelengths of the blue- and red-emitting CdSeS/ZnS QDs as they are assembled on the silica substrate (regions I and III) are 450 nm and 645 nm, respectively, as shown in Figure 1d.

### 3.1. Oscillatory Temporal Dynamics in PL Emission

**Figure 2:**
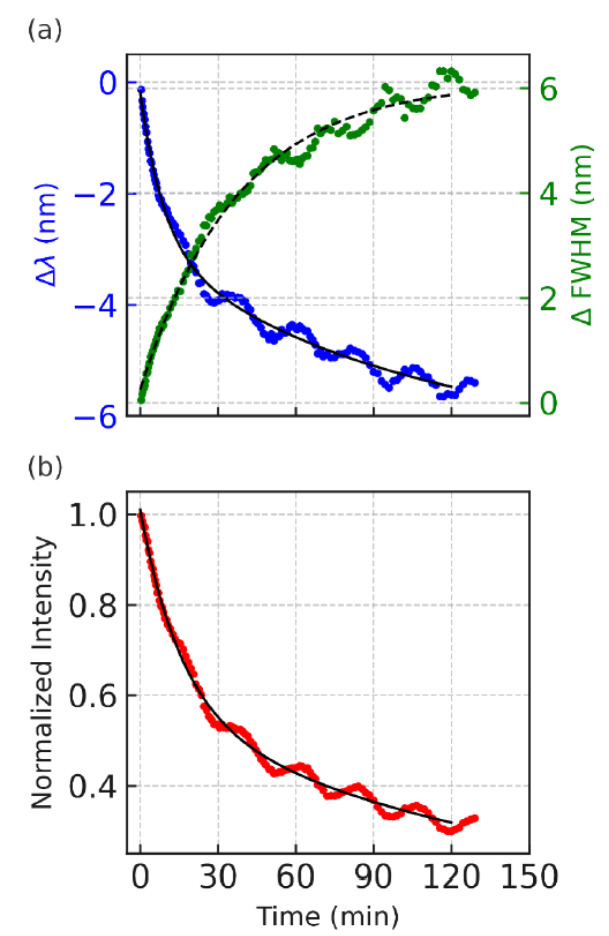
Temporal evolution of oscillatory photoluminescence (PL) emission in a stacked QD645/QD450 double-layer monolayer under continuous 532 nm laser excitation. Data are extracted from region II of the bilayer schematic shown in Figure 1a. (**a**) PL peak wavelength shift of QD645 (blue dots) and change in FWHM (green dots) as a function of time. (**b**) Normalized PL intensity of QD645 (red dots) over the same period. Both QD645 peak shift and intensity exhibit oscillatory decay behavior, well described by a biexponential model (solid black lines). In contrast, the FWHM dynamics follow a single-exponential trend (dashed black line) and display oscillations that are approximately 180° out of phase with the peak position and intensity variations. The shared *x*-axis represents time in minutes.

Figure 2 displays the time evolution of the photoluminescence (PL) characteristics from the top layer ternary CdSeS/ZnS quantum dot (QD) film with a nominal diameter of 12 nm under continuous 532 nm laser excitation. The upper panel in the figure (Figure 2a) simultaneously tracks the peak wavelength shift (blue trace) and full width at half maximum (FWHM; green trace), while the lower panel (Figure 2b) shows the PL intensity decay (red trace). All the measurements are plotted against time in minutes.

Over the course of the 120 min measurement, the PL emission characteristics exhibit a clear oscillatory behavior superimposed on their general trends. The emission peak wavelength shows a net blueshift, progressing up to approximately 10 nm, suggesting an increasing average recombination energy over time. Simultaneously, the FWHM undergoes gradual broadening, rising from near zero (change at initial time) to about 6 nm, indicating that the spectral profile becomes increasingly disordered or diverse. These spectral features mirror the underlying carrier dynamics and structural reorganization of the QD ensemble. However, excitation of the QD monolayers on the blue absorption edge results in drastic redshift in emission peak positions with time (see Section 3.2).

Most notably, both the wavelength shift and the FWHM trace display recurrent modulations with subtle amplitude. These non-random fluctuations reflect slow, damped oscillations around the mean trend line, suggesting a dynamic feedback loop in which the local band structure, strain field, or charge distribution periodically adjusts under continuous excitation. The oscillatory modulation in FWHM hints at transient narrowing and broadening cycles, which likely correspond to alternating phases of localized and delocalized recombination across spatially varying emission sites.

In the lower panel, the PL intensity likewise decreases over time but with visible quasi-periodic dips and partial recoveries, indicating that recombination efficiency is not monotonically declining. These dips appear to be phase-shifted relative to the spectral changes—periods of minimum intensity often align with blueshifted emission and a broader spectral width. This antiphase relationship supports a model in which recombination becomes less efficient as the system shifts toward higher-energy, more disordered states. Together, these oscillatory trends point to a coupled photophysical system in which trap carriers, stress relaxation, and band-edge modulation co-evolve under prolonged photoexcitation.

Each panel in Figure 2 displays experimental data (colored dots) overlaid with fitted exponential models (black or dashed lines) to isolate and quantify the relaxation dynamics governing the photoluminescence (PL) intensity, emission peak shift, and spectral broadening over time.

Accordingly, the data in Figure 2a also illustrates the peak wavelength shift fits well to biexponential decay. The overall blueshift trajectory suggests that recombination pathways shift from lower-energy, possibly trapped states toward higher-energy states over time. The fast component (τ_1_ ≈ 523 s) may reflect early-time carrier redistribution and the filling of shallower traps, while the slower component (τ_2_ ≈ 5541 s) is associated with gradual changes in the QD energy landscape, possibly due to photoinduced structural relaxation or band-edge modulation arising from accumulated excitation stress.

PL intensity is also modeled using a biexponential decay function (black solid line in Figure 2b), reflecting the presence of two distinct recombination pathways. The initial rapid decay (τ_1_ ≈ 953 s) likely corresponds to direct excitonic recombination, where photoexcited electrons and holes recombine efficiently in well-passivated QDs. The slower component (τ_2_ ≈ 8821 s) is attributed to trap-assisted relaxation or long-lived carrier retention, where charge carriers are transiently localized in sub-bandgap states or migrate across energetically disordered regions before recombination. This long tail is consistent with the persistent low-level emission observed even after extended illumination, and it sets the timescale for structural and environmental changes within the QD layer.

In contrast, FWHM broadening is best described by a single-exponential growth model (black dashed line Figure 2b) with a decay constant of approximately τ ≈ 2231 s. The monotonic increase in the spectral width suggests that the emission arises from an increasingly diverse set of recombination sites, either due to the progressive delocalization of carriers or the activation of QDs with slightly different bandgap energies. Unlike the PL intensity and wavelength shift, no significant secondary component was needed to capture the broadening trend, indicating that a single, slow process—likely involving the gradual introduction of energetic disorder—is sufficient to explain the FWHM evolution.

Collectively, the exponential fitting in Figure 2 disentangles the decay and broadening behavior of the PL signal into physically meaningful timescales. These fits confirm that multiple relaxation pathways govern the optical behavior of the ternary QD layer, and that structural and energetic reorganization unfolds over tens of minutes to hours, underpinning the damped oscillatory modulations observed. These findings reinforce the role of internal feedback mechanisms—such as carrier migration, trap cycling, and stress modulation—as key drivers of temporal and spectral variability in this system.

### 3.2. Photoluminescence Dynamics Under 405 Nm Excitation

**Figure 3:**
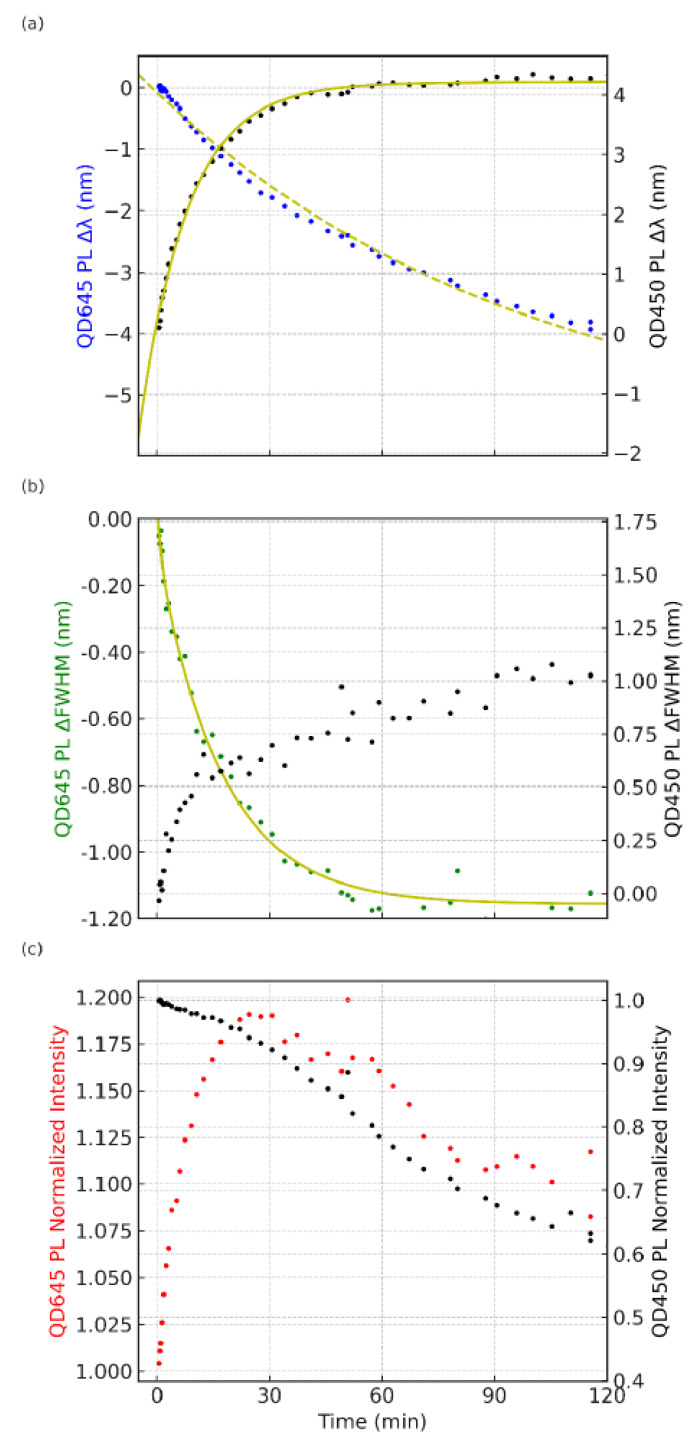
Temporal photoluminescence (PL) dynamics of a stacked QD645/QD450 double-layer system under continuous excitation at 405 nm, which corresponds to the blue absorption edge of QD450. Panel (**a**) shows the evolution of the QD645 emission peak wavelength shift (blue dots, left *y*-axis) and QD450 emission peak wavelength shift (black dots, right *y*-axis) as a function of time. Panel (**b**) presents the temporal change in the full width at half maximum (FWHM) of the PL emission peaks for QD645 (green dots, left *y*-axis) and QD450 (black dots, right *y*-axis), with a biexponential growth model overlaid on the QD450 data (solid yellow line). Panel (**c**) depicts the normalized PL intensity changes in QD645 (red dots, left *y*-axis) and QD450 (black dots, right *y*-axis) over time. The excitation wavelength (405 nm) is expected to simultaneously excite both QDs, leading to distinct PL dynamics compared to previous measurements with 532 nm excitation.

When the bilayer is excited at 405 nm (on the blue absorption edge of QD450), both QD species are directly excited, yielding simultaneous emissions from the QD450 and QD645 layers. Figure 3 shows that under this excitation, the two PL peaks exhibit markedly different temporal trends compared to the 532 nm case. The QD450 emission (black dots, Figure 3a) gradually redshifts by several nanometers over the course of the experiment, accompanied by a modest increase in its FWHM (black dots, Figure 3b) and a slow, steady decrease in its PL intensity (Figure 3c). In contrast, the QD645 emission (blue dots, Figure 3a) undergoes a slight blueshift with time and even shows a mild spectral narrowing (green dots, Figure 3b), while its intensity (red dot, Figure 3c) rises slightly during the initial illumination. Notably, the pronounced oscillatory modulations in the peak position and intensity observed under 532 nm excitation are largely absent with 405 nm excitation. Instead, the PL evolution proceeds in a smoother, monotonic fashion for both QDs, indicating a distinct photoluminescence response when both layers are excited concurrently. These contrasting dynamics highlight how changing the excitation wavelength fundamentally alters the emission behavior of the bilayer, with the 405 nm excitation condition producing a different, more steady-state photophysical regime compared to the oscillatory dynamics seen under 532 nm excitation.

The emergence of damp oscillations and a sustained blueshift in the PL response of layered ternary quantum dot (QD) films presents a deviation from canonical photoluminescence behavior under continuous excitation. These findings suggest the presence of underlying feedback mechanisms or slow dynamic processes that modulate the optical properties over time. Several hypotheses may account for these observations, though each remains incomplete.

While several hypotheses including potential gradients, strain relaxation, internal electric fields, and trap-state cycling could plausibly account for the observed PL oscillations, their mechanisms remain speculative and experimentally unverified. Using Vegard’s law we have calculated the lattice constant for the alloyed QDs and determined a lattice mismatch of ≥8%, which imply considerable lattice strain at the core/shell interface. This is known to promote interatomic diffusion and partial alloying during shell growth.

To isolate the role of compositional asymmetry and interfacial coupling, we conducted control experiments using symmetric and binary QD bilayer systems under identical conditions as shown in Section 3.4.

### 3.3. Control Experiment

**Figure 4:**
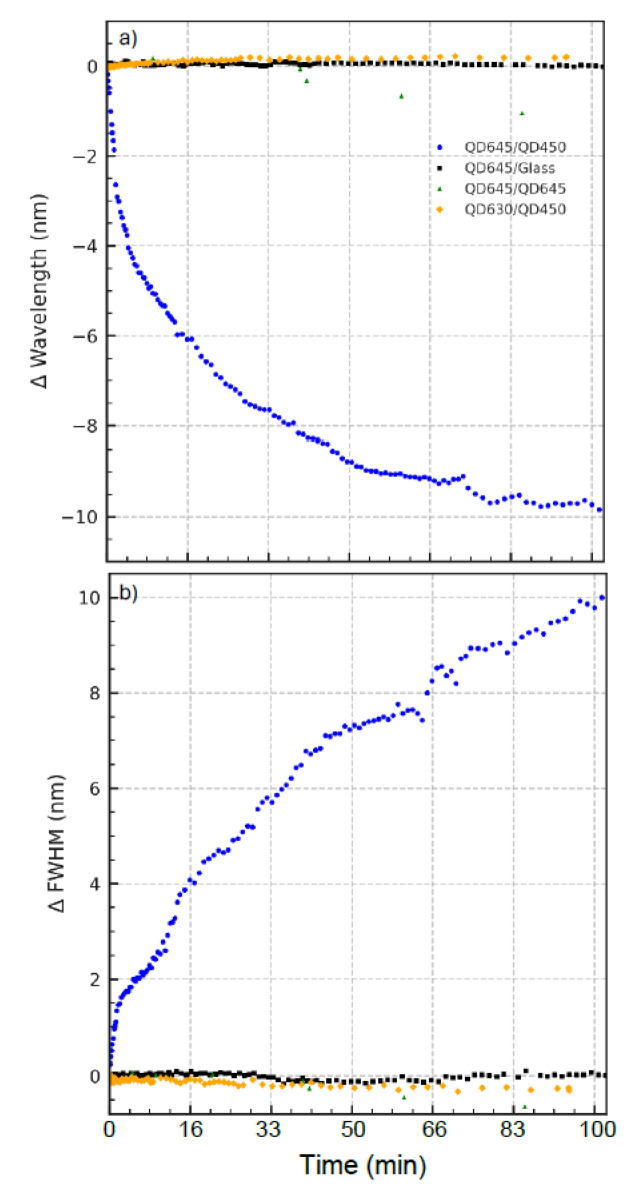
Control experiments for QD bilayers under 532 nm excitation. (**a**) Blue dots: QD645/QD450; black squares: QD645/glass; green triangles: QD645/QD645; and orange diamonds: QD630/QD450. Panel (**a**) shows peak wavelength shifts over time; (**b**) displays the corresponding change in FWHM.

To substantiate the hypothesis proposed in Figure 2 and Figure 3, namely, that the temporal oscillatory behavior in PL emission arises from interfacial processes between QD layers of differing composition, we performed control experiments using alternate quantum dot configurations. The results are summarized in Figure 4a,b and Supporting Information Figure S2a–c.

Figure 4a displays the time-dependent shift in the PL peak wavelength under continuous 532 nm excitation for four different QD film systems. The heterostructured QD645/QD450 bilayer (blue traces), which combines a ternary CdSeS/ZnS (QD645) top layer with a CdSeS/ZnS (QD450) bottom layer, exhibits a clear oscillatory blueshift—consistent with compositional evolution and bandgap modulation over time. In contrast, all three control systems exhibit negligible wavelength shifts throughout the same duration. The QD645/glass (black squares, Figure 4a) is an LB monolayer CdSeS/ZnS QD fabricated directly on a substrate. The QD645/QD645 (green triangles) is a symmetric LB bilayer. In contrast, the QD630/QD450 (orange diamonds) is an asymmetric LB bilayer formed by sequential deposition of two different quantum dots: a monolayer of CdSe/ZnS (QD630) LB film on top of a monolayer LB film of CdSeS/ZnS (QD450). This indicates that neither prolonged illumination nor substrate effects alone can account for the spectral behavior observed in the asymmetric bilayer, underscoring the necessity of compositional asymmetry and inter-QD contact for triggering dynamic interfacial processes.

Figure 4b reinforces this conclusion by examining the evolution of the full width at half maximum (FWHM) of the PL emission spectrum, plotted as the change relative to its initial value. As clearly observed in Figure 2, the QD645/QD450 film shows a gradual and oscillatory increase in the ΔFWHM, consistent with an increasing heterogeneity in recombination sites or evolving electronic disorder across the film. Meanwhile, the control systems maintain stable spectral widths, indicating that the observed broadening in the test system arises from interfacial structural reorganization, not general photobleaching or optical instability.

Figure S3 (Supporting Information) shows normalized PL intensity trends under continuous 532 nm excitation. The asymmetric QD645/QD450 bilayer exhibits a characteristic oscillatory decay, mirroring the spectral dynamics seen in Figure 4a,b. In contrast, all the control systems show an increase in PL intensity over time, albeit to varying degrees. This rise is minimal in QD645/QD645 and QD630/QD450 but pronounced in the QD645/glass sample, suggesting a strong photobrightening effect.

Interestingly, while the QD645/QD645 bilayer appears stable in terms of the peak wavelength (Figure 4a), its FWHM (Figure 4b) exhibits a slight narrowing trend over time. This subtle but reproducible behavior may reflect interlayer energy transfer or self-organization processes that enhance spectral uniformity, even in the absence of compositional asymmetry. Such narrowing is absent in other controls, indicating that interlayer communication in symmetric films, though weaker, may still lead to measurable spectral refinement over time.

Together, these control experiments validate the central claim that the PL oscillations and spectral changes reported in Figure 2 and Figure 3 are not universal effects of illumination or film fabrication but are instead the result of strain-induced, compositionally driven interfacial diffusion and dynamic bandgap restructuring in the asymmetric bilayer system.

### 3.4. Laser Power Dependence

**Figure 5:**
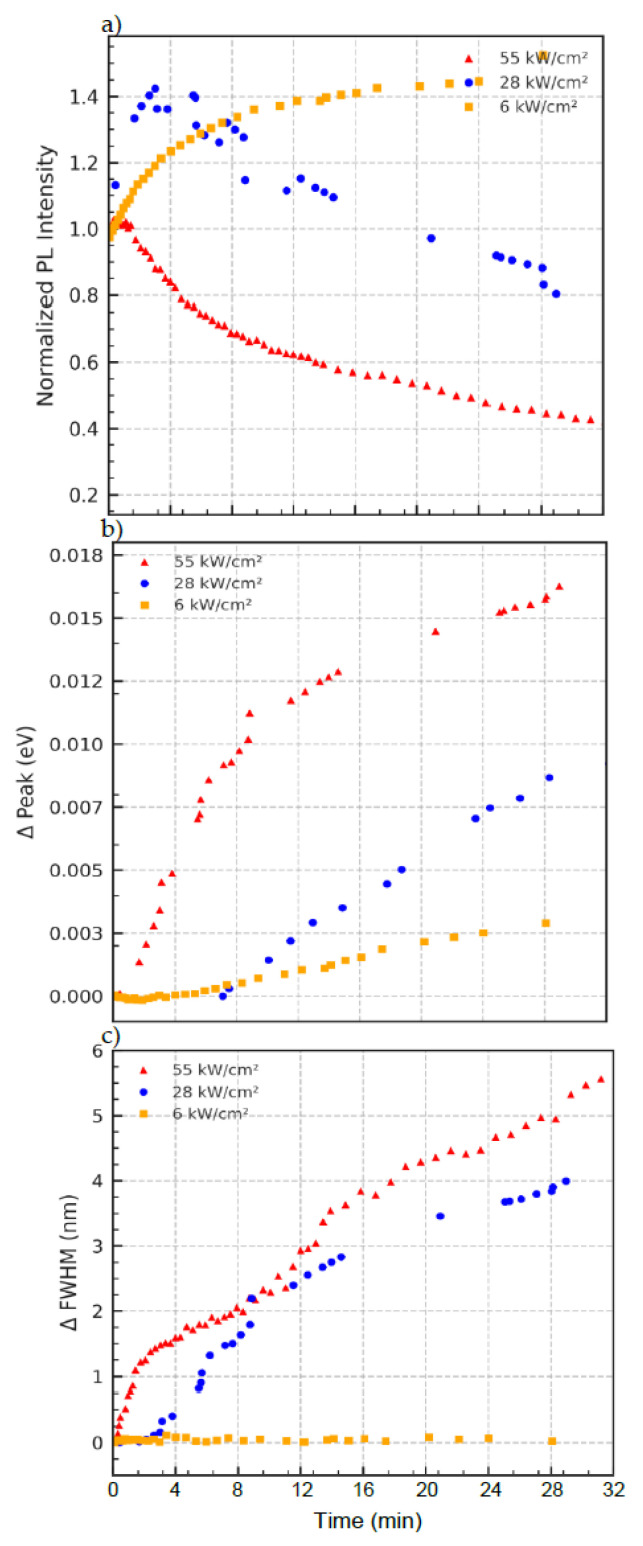
Excitation power-dependent evolution of PL characteristics from QD645/QD450 bilayers excited at 532 nm. (**a**) Red triangles, blue circles, and orange squares represent PL intensity for 55, 28, and 6 kW/cm^2^, respectively. (**b**) Peak emission energy shift (in eV) plotted with the same color/symbol coding. (**c**) Change in FWHM for the same power levels, using identical symbols.

The data in Figure 5a exhibits power-dependent PL intensity behavior. At low power (6 kW/cm^2^), the intensity slightly increases over time, suggesting mild reorganization or trap passivation. At high power (55 kW/cm^2^), the intensity decays more rapidly, consistent with photobleaching and enhanced nonradiative recombination pathways. Figure 5b depicts progressive blueshift in the emission peak, particularly at higher excitation powers. Figure 5c shows that spectral broadening is most pronounced at high excitation power. The ΔFWHM increases steadily with time, indicating increased energetic disorder, due to structural relaxation and interdot interaction. At lower powers, the FWHM remains more stable. However, the data in this trend supports a mechanism involving compositional redistribution (e.g., Se/S exchange) and bandgap widening, driven by strain and atomic diffusion between coupled quantum dots.

## 4. Discussion

In our Langmuir–Blodgett (LB)-assembled bilayers of ternary CdSeS/ZnS quantum dots (QDs), we observe striking oscillatory photoluminescence (PL) behavior under continuous excitation (Figure 2 and Figure 3). Rather than a simple photobleaching or monotonic shift, the PL intensity and peak wavelength fluctuate periodically over time. For example, the PL intensity rises and falls in an oscillatory fashion, and the emission peak of the bilayer alternately redshifts and blueshifts in successive cycles. Such oscillations are unprecedented in static QD films and point to active photophysical and photochemical processes occurring within the LB film during illumination.

This unusual behavior can be understood by considering the unique interlayer interactions enabled by the LB assembly. The LB technique produces extremely close-packed QD monolayers, and when two different QD monolayers (QD645 and QD450) are sequentially deposited to form a bilayer, the QDs from opposing layers come into intimate contact at the interface. Crucially, the LB process likely compromises the native surface ligands, bringing QD surfaces closer than in solution or thick films. It is known that exposing QDs to polar environments or short-chain solvents can strip away long-chain ligands, dramatically reducing interdot distances and quenching PL [22]. In our LB films, a similar effect is expected: the compression and transfer steps can displace or disorder the oleate ligands, allowing direct QD–QD contacts across the bilayer interface. Within a single monolayer, by contrast, neighboring QDs remain separated by whatever ligand shell remains, limiting any atomic exchange or charge transfer to relatively weak dipolar couplings. Thus, intralayer interactions are minimal, whereas interlayer interactions are pronounced in the bilayer. The experimental fact that oscillatory PL emerges *only* in the mixed bilayer (Figure 2 and Figure 3) and not in single-component films underscores that this effect originates from an interface-specific phenomenon introduced by the LB assembly.

At the interface between the two QD monolayers, we propose that a unique atom interchange process takes place, driven by the compositional asymmetry between the contacting QD645 and QD450 nanocrystals. Although both QD types are chemically similar alloyed CdSeS/ZnS heterostructures, their different emission wavelengths indicate different internal compositions and size (e.g., QD645 likely has a larger, more Se-rich core, whereas QD450 has a smaller, more S-rich core). When these dissimilar QDs are pressed into contact (with minimal ligand barriers), a thermodynamic driving force exists for atomic interdiffusion at the interface. In essence, the two QDs strive to reach a common composition at their juncture—a process akin to an alloying or cation/anion exchange localized at the contact region. We hypothesize that chalcogen exchange (Se↔S) or cation mixing (Cd↔Zn) occurs across the interface, altering the core compositions of both QDs in the contact pair. This photoinduced interdiffusion is plausible, given the energy input from the laser and the close proximity of the crystals. Analogous diffusion processes are well documented in core/shell QDs under thermal or photonic stimulus. For instance, under elevated temperatures, Zn^2+^ has been observed to diffuse from a ZnS shell into a CdSe-based core, leading to a continuous blueshift of the emission peak [23]. Such interdiffusion is often accompanied by lattice strain effects; as the shell material mixes into the core, the lattice mismatch is reduced, which can further shift the bandgap [23]. Indeed, in situ heating of a CdSe–CdS–ZnS core/multishell system causes Zn and Cd intermixing between layers, transforming the structure into an alloyed CdZnSe/CdZnS and changing its optical output [24]. By parallel, in our LB bilayer, the initial contact between a CdSeS-rich QD645 and a CdSeS-lean QD450 induces a “change–equilibrium–change” sequence: a rapid atomic rearrangement (change) at the interface as the two QDs partially exchange constituents, a temporary equilibrium when their interface compositions balance, and subsequent changes if new driving forces arise (e.g., further illumination or heating). This sequence will manifest as an oscillatory PL if each stage alters the emission in opposite directions. Notably, an initial intermixing could redshift the blue QD’s emission (by adding Se or increasing its size) while blueshifting the red QD’s emission (by adding S or reducing quantum confinement), bringing their peak wavelengths closer. If the process overshoots or reverses (for example, due to stress relaxation or reversible ligand reattachment), the peaks might then diverge again, producing a cyclical shift. The net result is a photoinduced oscillation in the optical properties, as observed.

The oscillatory PL intensity can be rationalized in a similar framework. Initially, the close packing and partial ligand stripping in the bilayer likely introduce nonradiative surface defect sites, causing an abrupt PL drop (first “valley” in the oscillation)—consistent with reports that removing native X-type ligands sharply reduces QY [22]. However, as interfacial atoms exchange and a more favorable bonding configuration sets in, some defects may be annealed out or new radiative recombination pathways might form, leading to PL recovery (“peak” in intensity). This mirrors behaviors seen in other QD systems where PL can darken then brighten under sustained illumination depending on surface chemistry [25]. For example, partial oxidation or alloying can initially trap excitons (diminishing PL) but subsequently passivate deep traps (recovering PL) [26]. In our case, the first atomic rearrangement at the QD–QD interface might alleviate strain or neutralize dangling bonds, temporarily restoring PL intensity. Subsequent cycles of intensity decrease, and an increase could arise from repeating structural/chemical oscillations, for instance, recurrent formation and healing of interfacial trap states. It is conceivable that local strain plays a role in this feedback: the lattice mismatch between QD645 and QD450 imposes stress at the interface, which could drive further atom exchange or defect creation to relieve that strain. In epitaxially fused QD superlattices [27,28], it is known that the “necks” connecting QDs introduce new electronic states and regions of different bandgaps [29]. Those neck regions, under strain, have distinctly larger bandgaps than the dot interiors, creating potential barriers that facilitate strong coupling between dots [29]. By analogy, the interfacial region in our bilayer behaves as a “neck” between two different QDs—any lattice strain or compositional gradient there could modulate carrier localization and recombination rates. Initially, the strain (and associated defects) might quench the PL [24]. But as atoms interchange to form a more compositionally graded interface (similar to inserting a graded CdS layer to mitigate CdSe/ZnS strain), the strain is partly relieved and the trap density drops, boosting the PL. This interplay of strain-driven defect formation and strain relief through interdiffusion could naturally lead to oscillatory PL output as the system finds a dynamic balance [30].

Control experiments (Figure 4) reinforce that the observed oscillatory photoluminescence dynamics are unique to the compositional and structural asymmetry of the QD645/QD450 LB bilayer. Neither symmetric LB bilayers of QD645/QD645 nor mixed bilayers of binary CdSe QD630 with QD450 exhibit such behavior, indicating that both alloyed composition and interlayer heterogeneity are critical. These findings suggest that strain-coupled interfacial atom exchange—enabled by compromised ligand coverage during LB assembly—drives the reversible bandgap modulation underlying the observed photophysical oscillations.

The experimental data in Figure 5 reveal that laser power strongly influences the dynamic optical response of the layered CdSeS/ZnS quantum dot (QD) films. At low excitation intensity (6 kW/cm^2^), minimal changes are observed in both the emission peak position and FWHM, and the photoluminescence (PL) intensity remains relatively stable. As the excitation power increases to 28 kW/cm^2^ and 55 kW/cm^2^, the system exhibits enhanced blueshifting of the PL peak and significant spectral broadening—both of which are classic hallmarks of thermally activated processes.

The PL intensity trends (Figure 5a) reinforce this interpretation: At low laser power, a modest increase in PL output is observed, likely due to trap passivation or photoactivation of emissive states. In contrast, under high-power excitation, the PL decay dominates, suggesting thermal quenching, irreversible trap formation, or structural reorganization of the QD layer. These observations are consistent with models where ligand disorder, interdot coupling, and thermal gradients co-modulate charge recombination dynamics [31].

In our LB-assembled QD heterostructures, where ligand shells are partially compromised and interlayer distances minimized, this thermal effect is compounded by enhanced interdot atomic communication. The power-dependent emission blueshift (Figure 5b) is indicative of dynamic compositional exchange at the interface—most plausibly Se migration out of QD645 and S enrichment from QD450—driven by photoinduced strain and asymmetric chemical potentials. Such an atom exchange has previously been reported in post-synthetic alloying or ripening of QDs under laser excitation [32] and is known to induce shifts in bandgap energies by modifying local stoichiometry.

In particular, the monotonic increase in FWHM under high-power excitation (Figure 5c) suggests thermal broadening of the electronic states within the QDs, a phenomenon attributed to elevated local temperatures in the film. This heating softens the energetic landscape of the nanocrystal ensemble, increasing electron–phonon interactions and enabling carriers to access a broader range of recombination pathways. This behavior is consistent with recent reports on quantum dot superlattices where thermally induced broadening and carrier delocalization were observed under moderate excitation power, particularly in epitaxially fused PbSe QD arrays [33].

Together, the power-dependent modulation of optical properties in layered QDs underscores a complex interplay of photoinduced strain relaxation, thermal disorder, and compositional diffusion—distinct from behaviors seen in monolayer or solution-phase QD systems. These insights not only enhance our understanding of QD films under optical stress but also highlight pathways for engineering dynamic optoelectronic responses in layered nanostructures.

## 5. Mechanistic Considerations and Future Outlook

The photophysical and photochemical behavior revealed by these LB-assembled QD bilayers is rich and complex. We propose that the LB film formation—by achieving close packing and compromised ligand passivation—enables a form of interlayer “communication” between QDs that is absent in well-passivated, isolated QDs. Through this interlayer coupling, one QD can influence its neighbor’s structure and emission via direct atomic exchange or strain transfer, a phenomenon reminiscent of coupled quantum dot superlattices where lattice distortions and electronic states propagate across particles [34,35]. In our system, the compositional asymmetry between the two QD species provides the driving force for an interface reaction: the QD pair essentially acts to reduce the free energy by evening out composition gradients (an entropic and enthalpic gain) at their juncture. Lattice contraction and enhanced interdot electronic coupling [36], as a result of ligand removal, is in agreement with our observations of emission dynamics in the LB films.

We acknowledge that we do not yet have a comprehensive model that quantifies this oscillatory PL behavior. The interplay of ligand dynamics, atomic diffusion, trap state formation/annealing, and thermal feedback is inherently complex. The observations, however, open up several avenues for future investigation. For instance, time-resolved spectroscopic studies (e.g., pump–probe or single-particle PL tracking) could shed light on whether the two QD components in the bilayer undergo synchronous emissivity changes or if one population leads the other. In situ structural characterization, such as TEM or X-ray scattering on a laser-illuminated bilayer, could directly reveal any interfacial alloy formation or periodic lattice expansion/contraction corresponding to the optical oscillations. Recent advances in operando X-ray diffraction have successfully captured light-induced structural dynamics in QD solids [37], offering a potential roadmap for such experiments. Our findings suggest that photo-annealing (via laser heating) in a hetero-QD bilayer can accomplish a similar feat in a highly localized, cyclic manner. There are also intriguing connections to strain engineering in QDs: imposing asymmetric strain on core/shell QDs has been shown to modify their electronic structure and eliminate certain nonradiative pathways [38,39]. In a colloidal bilayer, the strain generated at a heterointerface might shift energy levels or activate new recombination routes, contributing to the oscillatory output.

## 6. Conclusions

In summary, the oscillatory PL behavior of the CdSeS/ZnS QD bilayers is a photoinduced phenomenon enabled by the unique structural milieu of the LB film. The LB assembly provides the tight interlayer contact and partial ligand removal needed for direct QD–QD interactions, while the compositional difference between the two QD types drives a dynamic change–equilibrium–change process at the interface. The result is a self-modulating optical response: the bilayer’s emission toggles through different states (different intensities and peak positions) as it absorbs energy. We attribute this to a cycle of atomistic and defect-state transformations—likely involving interdiffusion (atom exchange) to relieve strain and align compositions. The experimental evidence (oscillations only in mixed bilayers, sensitivity to laser power, and the role of LB-induced ligand manipulation) all supports this interpretation. Nevertheless, the exact mechanistic pathway remains to be fully elucidated. Our work motivates further mechanistic studies, for example, to determine the role of lattice strain vs. chemical reaction, to quantify the kinetics of the interchange, and to explore if the oscillation can be controlled (or quenched) by environmental parameters like temperature or atmosphere. Unraveling this mechanism will not only explain the present observations but also deepen our understanding of photochemical reactions in nanocrystal solids—an emerging frontier where nanoparticles communicate and transform under illumination. Ultimately, the ability to induce and control such interparticle interactions could lead to new approaches in optoelectronic devices and smart materials, where an external stimulus (light or heat) actively tunes material properties in real time. Our findings, though currently without a complete model, lay the groundwork for these exciting future directions.

## Data Availability

Data are contained within the article and Supplementary Materials.

## Supplementary Materials

The following supporting information can be downloaded at https://www.mdpi.com/article/10.3390/nano15141113/s1, Figure S1: (a) Left, transmission electron microscopy (TEM) image of CdSeS/ZnS quantum dots (QD450) with a scale bar of 20 nm. The quantum dots appear well-dispersed with slight agglomeration. Right, TEM image of CdSeS/ZnS quantum dots (QD645) with a scale bar of 10 nm. The quantum dots are more uniformly distributed. Statistical analysis of particle size yields an average diameter of 7.0 ± 0.6 nm based on 1320 particles for QD450 while average particle diameter of 10.2 ± 0.7 nm based on 7188 particles for QD645. (b). High-resolution transmission electron microscopy (HRTEM) image QD450 (left) and QD645 (right). The core diameter is measured to be 5.5 nm for QD450 and 8.0 nm for QD645. (c) EDS spectrum of QD450 (top) and QD645 to estimate the Se/S ratio based on integrated peak area. The signals used for the analysis are highlighted purple for Zn, green for Se, and yellow for S. Table S1: Individual particle diameters (in nm) measured from high-resolution TEM images for QD450 and QD645 samples. The table lists the diameters of individual quantum dots along with the calculated average diameter and standard deviation for each sample. Figure S2: Temporal evolution of photoluminescence (PL) properties of symmetric CdSeS/ZnS (QD645) Langmuir–Blodgett (LB) bilayer films assembled on CdSeS/ZnS (QD645) under continuous 532 nm laser excitation along with the asymmetric PL properties for comparison. Figure S3: shows normalized PL intensity trends under continuous 532 nm excitation. The asymmetric QD645/QD450 bilayer exhibits a characteristic oscillatory decay, mirroring the spectral dynamics seen in Figure 4a,b. In contrast, all control systems show an increase in PL intensity over time, albeit to varying degrees. This rise is minimal in QD645/QD645 and QD630/QD450, but pronounced in the QD645/Glass sample, suggesting a strong photobrightening resulting from passivation effect.
