# Supplementary material for: Light-Driven Quantum Dot Dialogues: Oscillatory Photoluminescence in Langmuir–Blodgett Films
Source: Nanomaterials (Basel). 2025 Jul 18;15(14):1113. doi: 10.3390/nano15141113 (PMC12300110; doi:10.3390/nano15141113)
Supplement: Supplementary file 1 [file nanomaterials-15-01113-s001.zip › nanomaterials-3708174-supplementary.pdf]

Supporting Information

Title: Light-Driven Quantum Dot Dialogues: Oscillatory Photoluminescence in Langmuir-Blodgett Films

*Tefera Entele Tesema**, Department of Chemistry, Prairie View A&M University, Prairie View, TX 77446, USA*

*tetesema@pvamu.edu

1. Structural Characterization of QD450 and QD645

Supporting information Figure S1 depicts the relative size of quantum dots.

a)

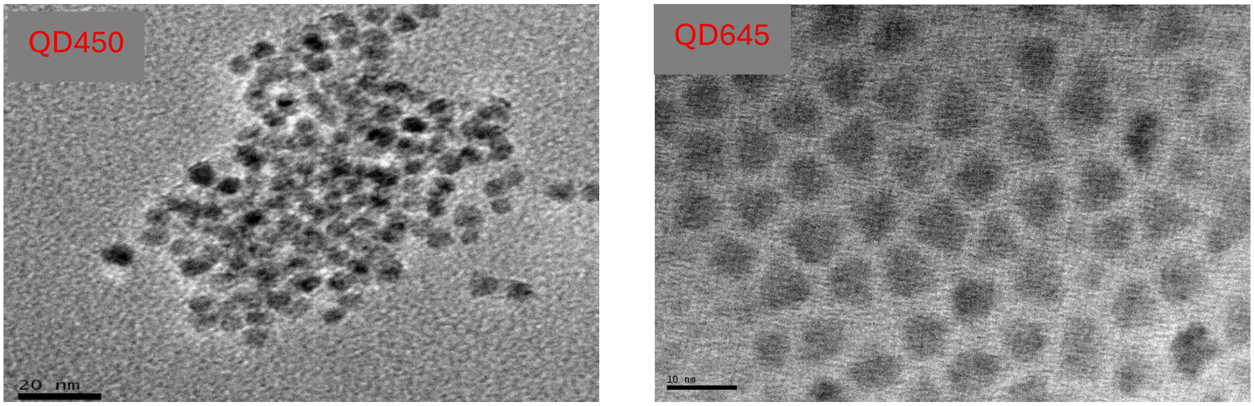

b)

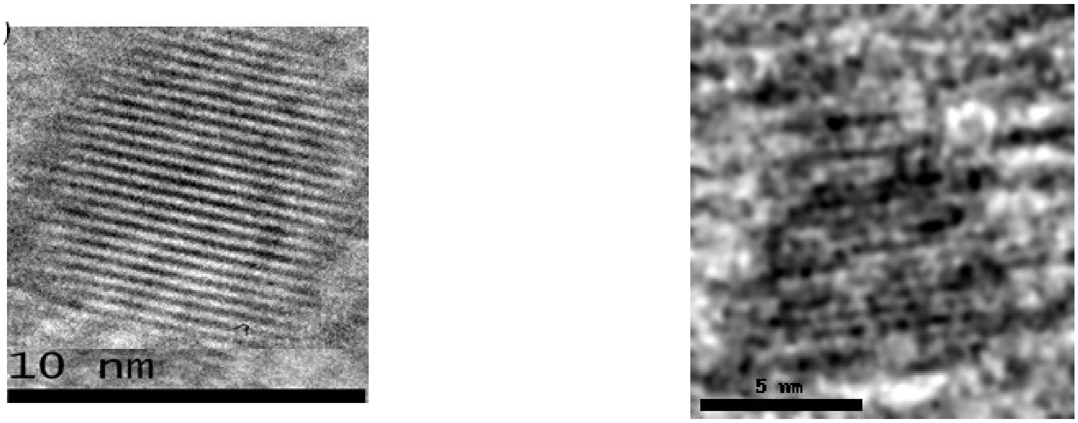

c)

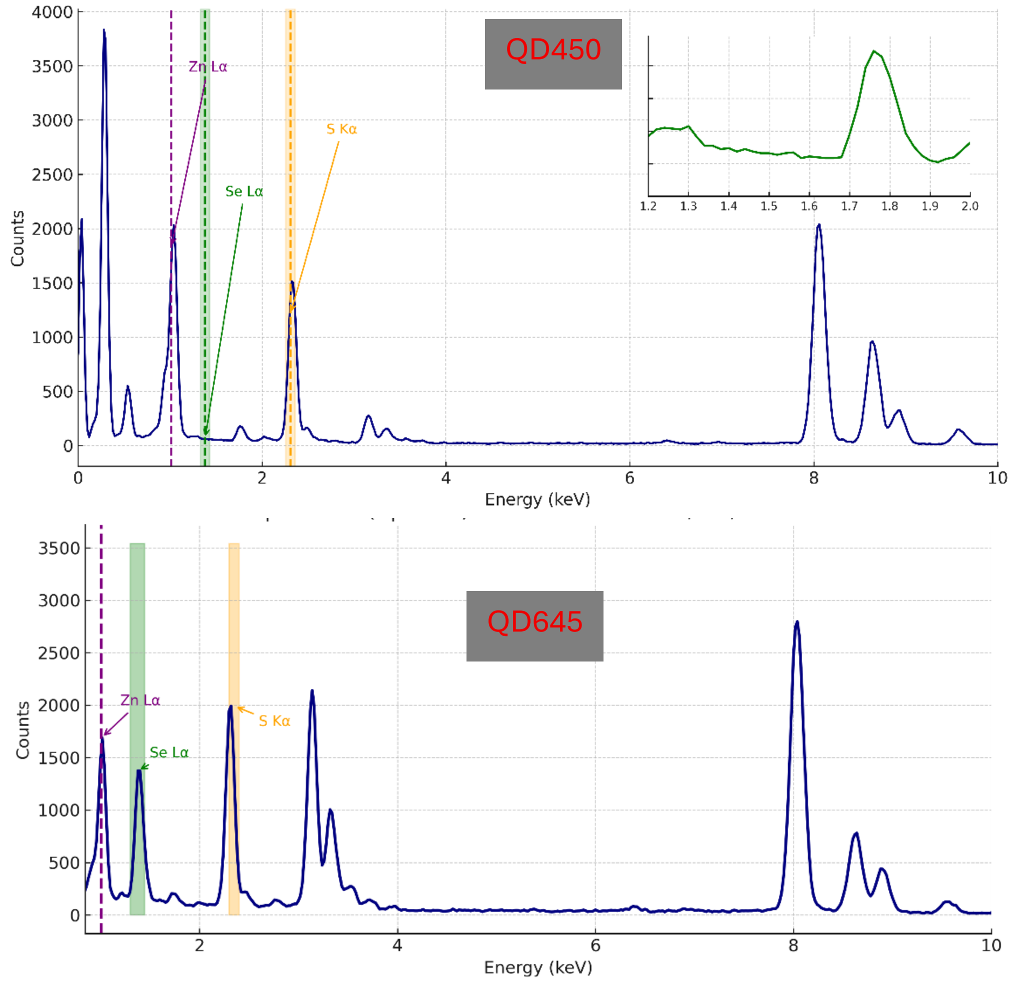

Figure S1.

(a) Left, transmission electron microscopy (TEM) image of **CdSeS/ZnS quantum dots (QD450)** with a scale bar of 20 nm. The quantum dots appear well-dispersed with slight agglomeration. Right, TEM image of **CdSeS/ZnS quantum dots (QD645)** with a scale bar of 10 nm. The quantum dots are more uniformly distributed. Statistical analysis of particle size yields an average diameter of 7.0 ± 0.6 nm based on 1320 particles for QD450 while average particle diameter of 10.2 ± 0.7 nm based on 7188 particles for QD645. **(b)**. High-resolution transmission electron microscopy (HRTEM) image QD450 (left) and QD645 (right). The core diameter is measured to be 5.5 nm for QD450 and 8.0 nm for QD645. (c) EDS spectrum of QD450 (top) and QD645 to estimate the Se/S ratio based on integrated peak area. The signals used for the analysis are highlighted purple fo Zn, green for Se, and yellow for S.

Table S1. Individual particle diameters (in nm) measured from high-resolution TEM images for QD450 and QD645 samples. The table lists the diameters of individual quantum dots along with the calculated average diameter and standard deviation for each sample.

CdSeS/ZnS (QD450)

#	Particle Size (nm)	#	Particle Size (nm)
1	8.59	33	7.08
2	7.86	34	7.47
3	7.03	35	6.41
4	7.38	36	6.62
5	6.65	37	7.66
6	6.45	38	7.35
7	7.31	39	6.94
8	8.16	40	7.02
9	7.81	41	7.47
10	7.42	42	7.18
11	6.84	43	6.81
12	6.98	44	6.44
13	7.13	45	6.08
14	6.17	46	6.22
15	6.57	47	8.05
16	7.25	48	7.98
17	6.87	49	6.02
18	7.61	50	6.23
19	7.61	51	6.42
20	6.56	52	6.55
21	7.55	53	6.72
22	6.05	54	6.45
23	7.19	55	7.34
24	6.1	56	6.89
25	7.11	57	6.44
26	7.85	58	6.53
27	6.21	59	6.79
28	7.68	60	6.17
29	8.5	61	6.54
30	7.3	62	7.82
31	6.31	63	6.03
32	6.44		
**Average (nm)**			**6.99**
**Std Dev.(nm)**			**0.65**

CdSeS/ZnS (QD450)

#	Particle Size (nm)	#	Particle Size (nm)
1	10.11	25	9.97
2	10.62	26	10.78
3	9.8	27	9.8
4	11.72	28	10.34
5	11.89	29	10.1
6	9.31	30	8.92
7	9.58	31	8.77
8	9.31	32	9.87
9	9.55	33	9.38
10	9.94	34	10.03
11	10.18	35	8.75
12	9.81	36	10.64
13	10.56	37	11.03
14	10.11	38	10.61
15	10.71	39	10.49
16	11.19	40	10.95
17	10.7	41	9.35
18	10.81	42	10.73
19	10.77	43	10.9
20	11.05	44	10.6
21	10.26	45	11.5
22	10.34	46	9.37
23	9.53	47	10
24	9.81		
	**Average (nm)**		**10.32**
	**Std. Dev.(nm)**		**0.70**

2. Photoluminescence properties of symmetric (QD645/QD645) and asymmetric (QD645/QD450) LB bilayers.

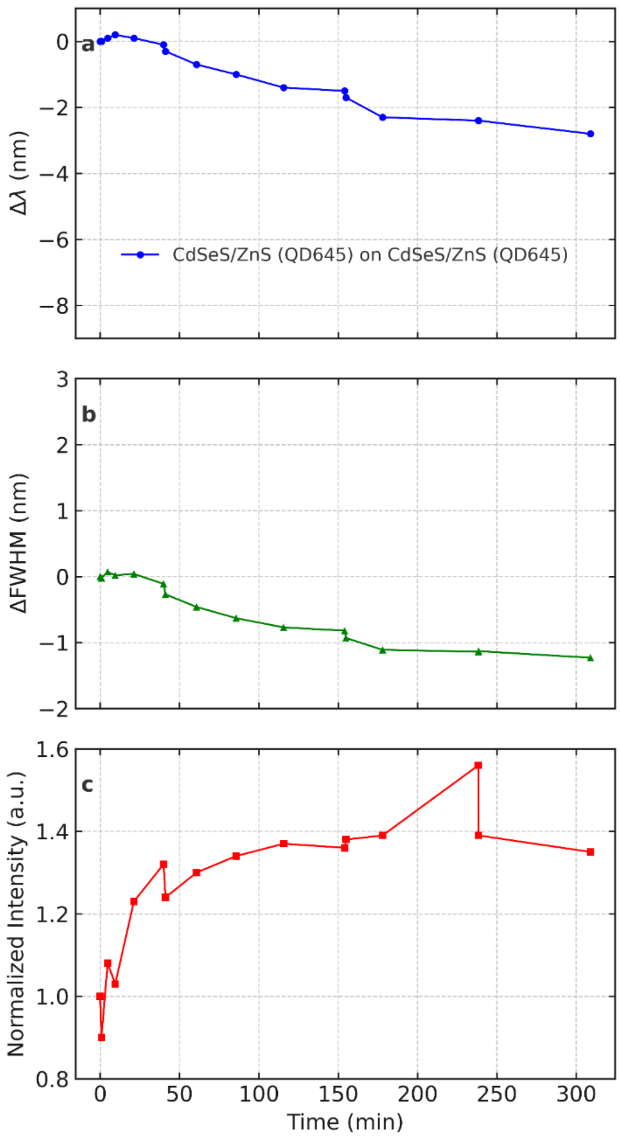

Figure S2.
Temporal evolution of photoluminescence (PL) properties of symmetric CdSeS/ZnS (QD645) Langmuir–Blodgett (LB) bilayer films assembled on CdSeS/ZnS (QD645) under continuous 532 nm laser excitation along with the asymmetric PL properties for comparison.

(a) Time-dependent shift in the emission peak wavelength (Δλ) recorded over the course of 300 minutes. The data are plotted as the change in peak position (Δλ, nm) versus time (min). Initially, the emission undergoes a rapid shift followed by a slow progression, suggesting a dynamic

reorganization or mild structural relaxation in the LB bilayer under photoexcitation. The legend "CdSeS/ZnS (QD645) on CdSeS/ZnS (QD645)" indicates the symmetric QD system without compositional asymmetry, serving as a control for the asymmetric bilayer experiments. The observed drift is relatively small (~2–3 nm), implying minimal photochemical or structural transformation in this symmetric configuration.

(b) Temporal evolution of the full-width at half maximum (FWHM) of the PL spectra (ΔFWHM) under identical excitation conditions. The FWHM shift (nm) is plotted versus time (min). The FWHM shows modest fluctuations though not significant it shows narrowing trends, further confirming the structural stability of the QD ensemble. Minor variations are attributed to slight surface rearrangements or local carrier redistribution rather than major compositional or atomic reorganization, as no interdot chemical potential gradient exists in the symmetric system.

(c) Evolution of the normalized PL intensity as a function of time. The normalized intensity (a.u.) is plotted against time (min). A gradual decrease in PL intensity is observed, characteristic of photobleaching behavior common in colloidal QD systems under prolonged illumination. The decay profile, however, lacks oscillatory features, supporting the hypothesis that the absence of compositional asymmetry and minimal ligand disturbance in the symmetric bilayer configuration prevents dynamic interfacial processes such as atom exchange or strain modulation, which are critical for the oscillatory behavior observed in asymmetric bilayer films.

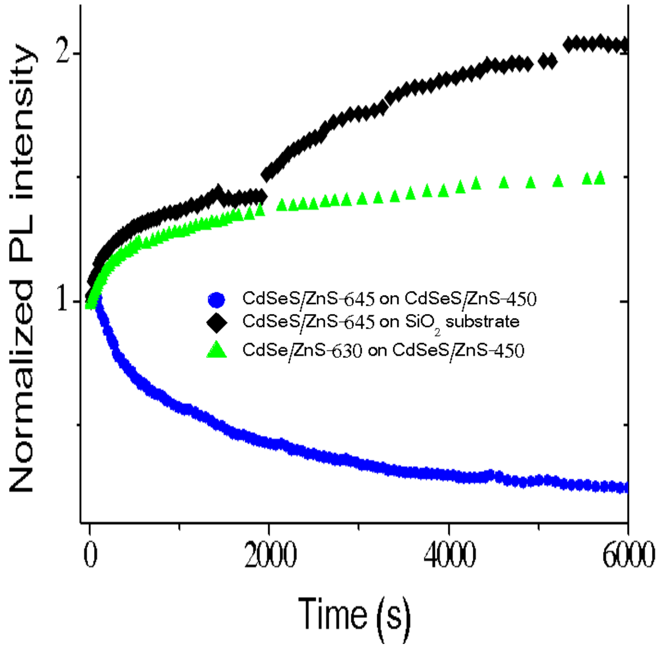

Figure S3 (**Supporting Information**) shows normalized PL intensity trends under continuous 532 nm excitation. The asymmetric QD645/QD450 bilayer exhibits a characteristic oscillatory decay, mirroring the spectral dynamics seen in **Figure 4a** and **Figure 4b**. In contrast, all control

systems show an increase in PL intensity over time, albeit to varying degrees. This rise is minimal in QD645/QD645 and QD630/QD450, but pronounced in the QD645/Glass sample, suggesting a strong photobrightening resulting from passivation effect.
